# Evidence that ribosomal protein bS21 is a component of the OLE ribonucleoprotein complex

**DOI:** 10.1080/15476286.2025.2491842

**Published:** 2025-05-05

**Authors:** Freya D. R. Wencker, Seth E. Lyon, Ronald R. Breaker

**Affiliations:** a Department of Molecular, Cellular and Developmental Biology, Yale University, New Haven, CT, USA; b Department of Molecular Biophysics and Biochemistry, Yale University, New Haven, CT, USA

**Keywords:** bTOR, extraribosomal function, extremophile, membrane, protein moonlighting, noncoding RNA, stress response, translation

## Abstract

OLE RNAs represent a large and highly structured noncoding RNA (ncRNA) class that is mostly found in Gram-positive extremophiles and/or anaerobes of the Bacillota phylum. These ~600-nucleotide RNAs are among the most structurally complex and well-conserved large ncRNAs whose precise biochemical functions remain to be established. In *Halalkalibacterium halodurans*, OLE RNA is involved in the adaptation to various unfavourable growth conditions, including exposure to cold (≤20°C), ethanol (≥3% [v/v]), excess Mg^2+^ (≥4 mM), and non-glucose carbon/energy sources. OLE forms a ribonucleoprotein (RNP) complex with the OLE-associated proteins A, B and C, which are known to be essential for OLE RNP complex function in this species. Bacteria lacking OLE RNA (Δ*ole*) or a functional OLE RNP complex exhibit growth defects under the stresses listed above. Here, we demonstrate that ribosomal protein bS21 is a natural component of the OLE RNP complex and we map its precise RNA binding site. The presence of bS21 results in a conformational change in OLE RNA resembling a k-turn substructure previously reported to be relevant to the function of the OLE RNP complex. Mutational disruption of the bS21 protein or its OLE RNA binding site results in growth inhibition under cold and ethanol stress to the same extent as the deletion of the gene for OLE RNA. These findings are consistent with the hypothesis that bS21 is a biologically relevant component of the OLE RNP complex under a subset of stresses managed by the OLE RNP complex.

## Introduction

**Figure 1.**
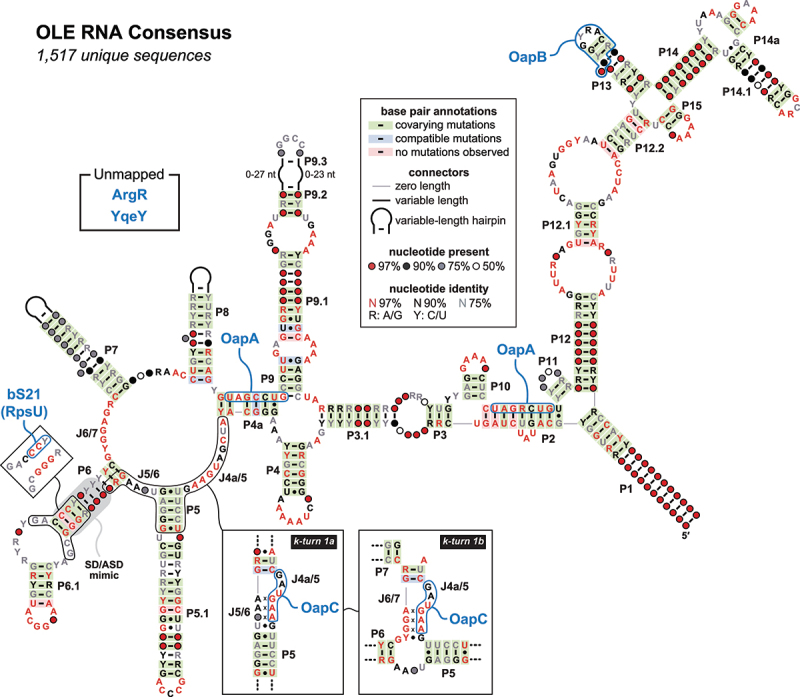
Consensus sequence and secondary structure model of OLE RNAs. This model is updated from a previous version [1] and is based on the alignment of 1,517 unique representatives from bacterial genomic and metagenomic sequences. Data supporting the OLE RNA bindings sites (blue enclosures) for OapA [12], OapB [14,15], and OapC [16] were reported previously. Data supporting the bS21 binding site is presented herein, including the identification of a mimic of the SD/ASD substructure (shaded grey). Three mutually exclusive, but apparently relevant, structures are depicted for the region encompassed by J4a/5 through the J6/7 nucleotides, including the alternative called k-turn 1a and k-turn 1b (boxed). Evidence for ArgR and YqeY binding (unmapped) remains unpublished.

Ornate, large, extremophilic (OLE) RNAs are a class of large noncoding RNAs (ncRNAs) found in anaerobic and/or extremophilic bacteria of the Bacillota (formerly Firmicutes) phylum [1]. OLE RNAs were initially identified by conducting comparative sequence analyses to search for novel structured ncRNAs in bacteria [2,3]. Members of this ncRNA class, each typically ~600 nucleotides in length, are among the most structurally complex and well-conserved bacterial large ncRNAs [4] identified to date (Figure 1). In addition, OLE RNAs represent the largest, most complex and widespread bacterial ncRNA whose biochemical function(s) remain to be established.

In the haloalkaliphile *Halalkalibacterium halodurans* [5,6], OLE RNA is a major component of a ribonucleoprotein (RNP) complex required for the bacterium to robustly respond to various fundamental stresses [1]. This bacterial species was chosen as a model organism for the study of OLE RNA because it is an extremophilic facultative anaerobe that naturally carries this ncRNA and because it can be grown under aerobic conditions with routine culture methods. Methods for the genetic manipulation of this species have also been developed [7,8], which enable the selective knockout or introduction of genes. Growth deficiency phenotypes for strains lacking a functional OLE RNP complex have been reported for the following stresses: cold (≤20°C); ethanol (≥3% [v/v]) or other short-chain alcohols [9]; Mg^2+^ (≥4 mM) [10]; non-preferred carbon/energy sources [11]. Given these diverse phenotypes and many other characteristics evocative of mTOR complexes found in eukaryotes, we have named this RNP complex ‘bTOR’ (bacterial Tasks OLE Regulates) [1]. In other words, we proposed that OLE RNA is part of a sophisticated RNP complex that participates in the sensing and mitigation of fundamental stresses, in part by regulating major physiological changes. OLE RNA is highly abundant even under normal growth conditions, and becomes the 5^th^ most abundant RNA transcript, excluding rRNAs and tRNAs, when cells are exposed to ethanol stress [9].

The published phenotypes of OLE RNA knockout cells [9–11], and others not yet reported, can be brought about by genetic disruption of the gene for OLE RNA (*ole*), or by the individual disruption of three other genes whose protein products are known to be necessary for the function of OLE RNP complexes in this species. These proteins, called OLE-associated proteins A, B, and C (OapA [3,12,13], OapB [13–15], and OapC [13,16], were identified as binding partners for OLE RNA using four distinct approaches. OapA was first identified because the *ole* and *oapA* genes colocalize in the genomes of most host species [1,3,12], suggesting the two gene products are functionally related [17,18]. OapB and OapC were first identified because mutations in the *oapB* and *oapC* genes appeared in genetic selections for *H. halodurans* mutants that disable the OLE RNP complex [13]. Strong evidence that OapB and OapC were OLE RNP components was also derived from the list of proteins that were purified via RNA ‘pull-down’ experiments [16] using a method called CHART [19]. Finally, implementation of a computational approach called phylogenetic profiling revealed that these three proteins are most often present in cells that carry OLE RNA [20], thus affirming the known functional links between these molecules.

The characteristics of these protein partners have helped guide hypotheses regarding the function of OLE RNA. For example, OapA is a 21-kDa transmembrane protein that appears to form a 2:1 OapA:RNA complex by binding nucleotides predicted to participate in base-paired regions P2 and P4a (Figure 1). These interactions are necessary for the particle to localize to the cell membrane [12]. Also, OapA shares certain sequence and structural characteristics with MpfA-family proteins [10,20], suggesting that OapA is involved in divalent metal ion transport. OapB (formerly YbzG) is a 11-kDa KOW [21] domain-containing protein of unknown function that strongly binds OLE RNA in the P13 stem-loop region [14,15]. OapC (formerly YbxF) is a 9-kDa homolog of the archaeal L7Ae protein [22] that has been shown [16] to induce the formation of a k-turn [23] RNA substructure within OLE RNA. Thus, OapB and OapC appear to assist in OLE RNA folding and structural switching, although other functions might also remain to be discovered.

Despite numerous findings regarding the biological roles of OLE RNA and its larger RNP complex, the precise molecular mechanisms of action of this particle remain elusive. Identification of additional protein partners, especially those with known biological and biochemical functions, would greatly benefit the effort to identify these mechanisms. We speculate that many additional protein components of the OLE RNP complex remain to be identified, although not all partners would be expected to be essential for the broadest functions of OLE-based bTOR devices. Indeed, long lists of possible protein partners of OLE RNA have already been assembled from genetic selections [10,11,13], CHART RNA pull-down experiments [16], and bioinformatic analyses [20].

A top candidate from the list of possible RNA binding partners revealed by CHART is the ribosomal protein bS21 (RpsU) [16]. bS21 is part of the small (30S) ribosomal subunit [24–26] that is required for efficient translation initiation [26–28], presumably by enabling binding of the anti-Shine-Dalgarno (ASD) region of the 16S rRNA to the Shine-Dalgarno (SD) sequence [29] in mRNAs. This is a particularly intriguing candidate because, if bS21 can be confirmed as a biologically relevant partner of the OLE RNP complex, then OLE RNA might exploit this interaction as a mechanism for sensing and managing fundamental cellular stresses relevant to translation. Most studies on bS21 have been carried out in *Escherichia coli* in which bS21 is essential [30]. Far less is known about the exact contribution of bS21 in translation in Gram-positive organisms such as *Bacillus subtilis* in which the protein is non-essential [31]. However, a *B. subtilis* strain lacking bS21 exhibits an unusual ribosome profile, impaired growth, and both cell division and motility defects suggesting an important role in translation [31,32].

Herein, we confirm that bS21 selectively binds to OLE RNA and we localize this binding site to the P6 substructure of the RNA (Figure 1). Notably, this region of the *H. halodurans* OLE RNA carries a perfect mimic of the canonical ASD sequence of 16S rRNAs from this same species. Disruption of the ASD mimic by mutation of OLE RNA prevents bS21 binding. Furthermore, we demonstrate that bS21 binding induces a structural change in OLE RNA that resembles the same k-turn conformation known [16] to be bound by OapC in vitro. Finally, either a *H. halodurans* bS21 knockout (KO) strain or a strain carrying an OLE RNA mutant lacking the ability to bind bS21 both exhibit growth phenotypes that match those observed for strains lacking a functional OLE RNP complex when exposed to cold or ethanol stresses. These findings strongly indicate that bS21 is a biologically relevant binding partner of OLE RNA in *H. halodurans*. This interaction provides a physical link between OLE RNA and a component of ribosomes, by which OLE RNP complexes could influence, or be influenced by, the process of translation.

## Results and discussion

### Ribosomal protein bS21 binds OLE RNA in vitro

Ribosomal protein bS21 was identified as a potential component of the OLE RNP complex in a previous RNA ‘pull-down’ study that detected proteins interacting with *H. halodurans* OLE RNA [16]. This protein was among the highest-ranking candidates identified under stringent wash conditions, along with the previously validated OLE RNP components OapB [13–15] and OapC [16]. To assess whether bS21 indeed interacts with *H. halodurans* OLE RNA (Supplemental Figure S1), bS21 protein from this same species was prepared by expression in *E. coli* for use in binding assays (Supplemental Figure S2). Filter-binding assays, using both nitrocellulose (protein binding) and nylon (RNA binding) layers, were used to establish the extents of RNA-protein binding with various 5' ^32^P-labelled RNA constructs. Although electrophoretic mobility shift assays (EMSAs) can inform on these binding interactions, these were not extensively used in this study because the small size of bS21 (6.8 kDa, 57 amino acids) compared with full-size OLE RNA (~193 kDa, 637 nucleotides) yields only subtle shifts in mobility upon complex formation (Supplemental Figure S3).

**Figure 2.**
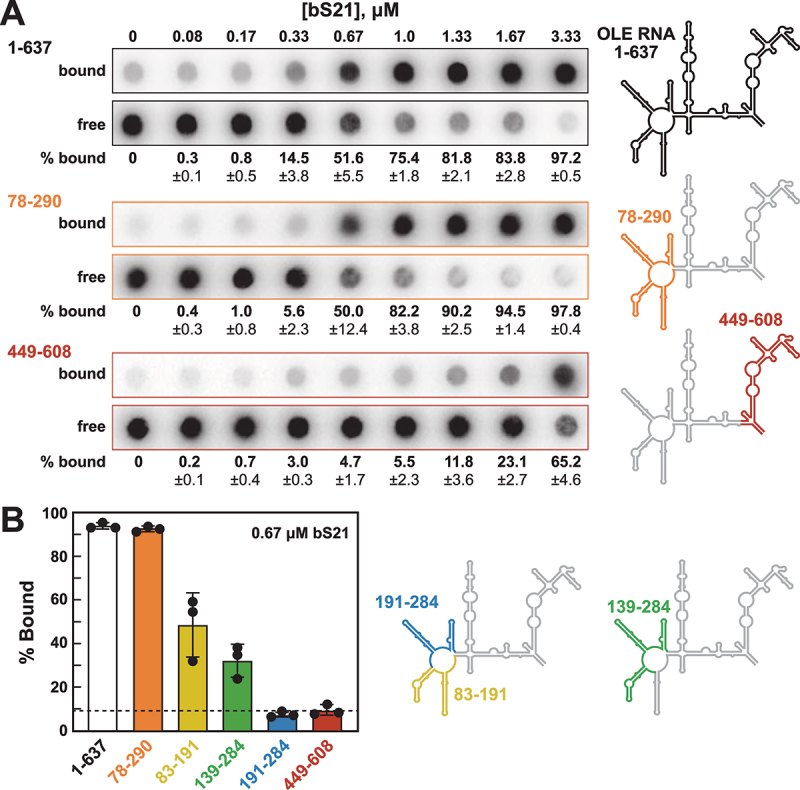
Ribosomal protein bS21 binds to OLE RNA in vitro. (A) Representative filter-binding assays with 5' ^32^P-labelled full-length OLE RNA (OLE_1–637_), and shortened constructs OLE_78–290_ and OLE_449–608_ (as highlighted to the right). Each paired dataset includes an autoradiogram of a nitrocellulose membrane (top row, ‘bound’) that captures RNA-protein complexes and a nylon membrane (bottom row, ‘free’) that captures RNAs that were not bound to protein and therefore passed through the nitrocellulose membrane. Percentage bound (% bound) values represent the average percentage of RNA bound to bS21 protein after subtraction of residual RNA remaining associated with nitrocellulose in the absence of added protein (see materials and methods for details). % bound and standard deviation values were computed based on three replicate assays. (B) Plot of the percent of trace amounts of 5' ^32^P-labelled RNA constructs bound to protein when incubated with 0.67 μM bS21 as determined by filter-binding assays. The horizontal axis labels indicate the various OLE RNA constructs. % bound values and standard deviation values are derived from three replicates.

As expected, based on the strength of this candidate OLE RNP component [16], bS21 robustly interacts with the full-length *H. halodurans* OLE RNA construct (OLE_1–637_) in vitro (Figure 2A, top panel). A protein concentration of 0.67 µM causes approximately half of the radiolabelled RNA to be retained by the nitrocellulose filter, which provides an estimate of the apparent dissociation constant (*K*_D_). Individual-length radiolabelled portions of OLE RNA were then evaluated to begin the process of mapping the site(s) of bS21 binding. For example, an OLE RNA segment spanning nucleotides 78 through 290 (OLE_78–290_) is also robustly bound by bS21 (Figure 2A, middle panel). In contrast, the OLE_449–608_ segment exhibits evidence of binding only when bS21 concentrations are very high (Figure 2A, bottom panel).

Similar assays with 0.67 µM protein and various OLE RNA segments yield results (Figure 2B) suggesting that a high-affinity binding site for bS21 resides in the P6 region of OLE RNA. However, an OLE RNA segment comprising only P6 flanked by the nucleotides in J5/6 and J6/7 was not robustly bound by bS21 (data not shown). These findings suggest that bS21 might rely on additional flanking regions to form productive contacts that improve its binding affinity to OLE RNA. Therefore, we chose to use the OLE_83–191_ segment for subsequent binding studies.

### bS21 binds a region of OLE RNA containing a mimic of the SD complex with 16S rRNA

**Figure 3.**
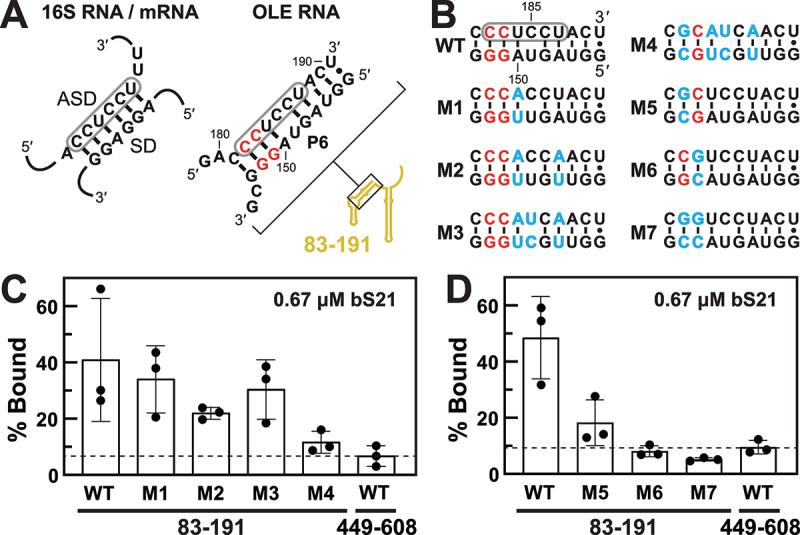
bS21 protein binds a region of OLE RNA that mimics the sequence and structure of a duplex formed between the *H. halodurans* 16S ribosomal RNA anti-Shine-Dalgarno (ASD) sequence and the matching Shine-Dalgarno (SD) sequence of an mRNA. (A) Left: schematic representation of a perfect ASD-SD duplex formed by the 3' end of *H. halodurans* 16S rRNA and the SD region of an mRNA, wherein the ASD sequence is encircled in grey. Right: sequence and predicted secondary structure of the *H. halodurans* OLE RNA P6 region (Supplemental Figure S1), wherein the ASD sequence match is encircled in grey. Red letters identify nucleotides that are conserved in 97% or more of the known OLE RNA representatives (Figure 1). The location of the P6 region within the OLE_83–191_ fragment is identified. (B) Sequence of the P6 region of wild-type (WT) OLE RNA and mutant constructs M1 through M7. Red letters identify highly conserved nucleotide positions and blue letters identify the sites and identities of the changes present in each mutant construct. (C) Plot of the percentage of 5' ^32^P-labelled OLE_83–191_ RNA constructs bound to bS21 at a concentration of 0.67 µM as determined by filter-binding assays. OLE_449–608_ serves as a non-binding RNA construct for comparison. The % bound and standard deviation values were generated based on three replicates. (D) Plots as described in C for additional RNA constructs.

After determining the tightest binding site for bS21 is in the P6 region, we noticed that this stem contains a sequence that perfectly matches the 3' tail of *H. halodurans* 16S rRNA (5'-CCUCCU) (Supplemental Figure S1), which is extensively complementary (or ‘anti’) to the Shine-Dalgarno (SD) sequences [29] of mRNAs. In *H. halodurans* OLE RNA, this ASD sequence is predicted to near-perfectly base-pair with a sequence in P6 that resembles an SD sequence (5'-AGUAGG), thereby forming a mimic of the structure formed when 16S rRNAs engage mRNAs via base-pairing to their SD sequences (Figure 3A). This similarity is highly relevant to our study because bS21 is known to bind near the engagement site between 16S rRNAs and mRNAs [24–26], and to affect translation initiation [26–29].

We hypothesized that bS21 binding to OLE RNA occurs in a manner similar to the interaction of bS21 with the 16S rRNA in the context of the ribosome. To assess this hypothesis, a series of mutant constructs of OLE_83–191_ were prepared and subjected to filter-binding assays with 0.67 µM bS21. For example, constructs M1 through M3 (Figure 3B) carry mutations that alter the sequences of the mimic SD and ASD segments, but that retain base-pairing potential of the original P6 duplex and avoid alterations at locations known to be most highly conserved in OLE RNAs. These three mutant constructs appear to be bound by bS21 to approximately the same extent as the WT OLE_83–191_ construct under the assay conditions used (Figure 3C). In contrast, construct M4, which carries the same mutations as M3 plus an inversion of a highly conserved G-C base-pair to a C-G base-pair (G152C and C182G), exhibits a substantial loss of bS21 binding.

Additional mutant constructs were prepared and tested to further evaluate the roles of the highly conserved G-C base-pairs in this region of P6. Constructs M5 and M6, each carrying a single inversion of a G-C base-pair exhibit substantial loss of bS21 binding (Figure 3D). Construct M7, which combines the mutations of M5 and M6, exhibits the poorest binding characteristics. These findings support our hypothesis that key determinants for bS21 binding reside in the SD-ASD-like duplex within P6. However, this data does not reveal if the most important nucleotides for bS21 binding are highly conserved in OLE RNAs for the purpose of binding to bS21, or for preserving the ability to form a functional OLE RNP complex in general.

**Figure 4.**
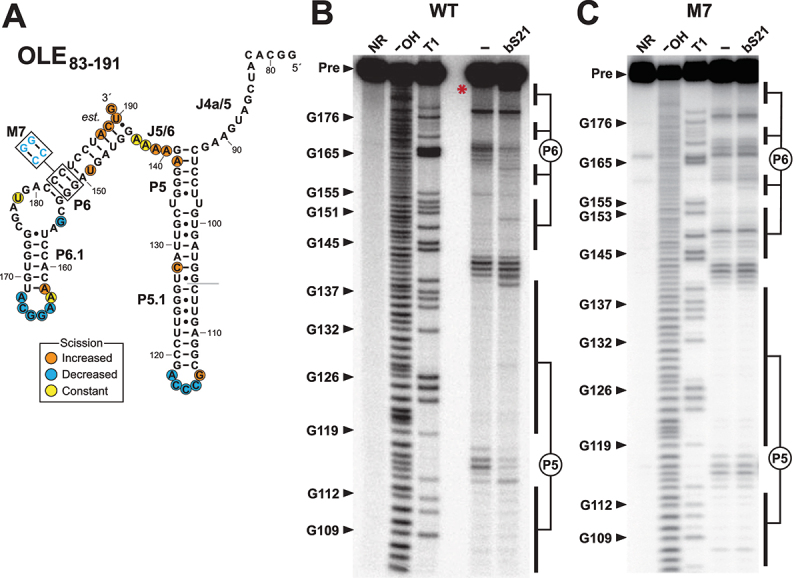
In-line probing assays of 5' ^32^P-labelled WT and M7 OLE_83–191_ RNA. (A) Sequence and secondary structure model for the WT and M7 OLE_83–191_ RNA constructs. Colored circles indicate characteristics of the in-line probing data for the WT RNA construct as derived from B, and ‘est’. indicates that the locations of the scission annotations are only estimated due to lower nucleotide resolution towards the 3' end of the RNA. The grey line denotes the start of the in-line probing data. (B) Autoradiogram of in-line probing assays conducted with the WT construct. Marker lanes denoted NR, ^−^OH, and T1 were loaded with RNA samples subjected to no reaction (reveals preexisting degradation products), partial alkaline digest (cleaves after all nucleotides), and partial digestion with RNase T1 (cleaves after G nucleotides). In-line probing reactions were conducted in the absence of added protein (‒) or in the presence of 1 µM bS21. Arrowheads identify full-length precursor (Pre) or various bands corresponding to select RNA fragments that have been cleaved after G nucleotides by RNase T1. Locations of regions corresponding to stems P5 and P6 are also indicated. Red asterisk identifies a region of prominent change in the band pattern upon the addition of bS21. (C) Autoradiogram of in-line probing assays conducted with the M7 construct. Annotations are as described for B. *Note*: the autoradiogram image for M7 has been vertically compresses to improve the ability to visually compare bands between WT and M7. The uncompressed image is presented in Supplemental Figure S6.

WT OLE_83–191_ (Figure 4A) and mutant RNA constructs M4 through M7, which exhibit partial or complete protein binding disruption, were also subjected to a structural analysis method called in-line probing [33,34] to determine if they retain local substructures formed by the original OLE RNA molecule. In-line probing exploits the inherent instability of unstructured RNA regions to reveal general structural features. The WT construct exhibits a clear pattern of spontaneous RNA cleavage products (Figure 4B) that is consistent with the formation of the P5 and P6 hairpins as proposed for full-length OLE RNA [16] (Supplemental Figure S1). The addition of bS21 induces changes in this pattern, indicating that protein binding causes folding changes most prominently in the loops of P5 and P6, and at the 3' terminus of P5 (Figure 4A). Although the M7 construct produces a nearly identical in-line probing pattern as WT, bS21 addition does not trigger changes in this pattern (Figure 4C, Supplemental Figure S4), consistent with the fact that M7 was not observed to bind to bS21 in dot-blot assays (Figure 3D).

Constructs M4, M5, and M6 yield in-line probing patterns that are consistent with the formation of the P5 hairpin but only an incompletely formed P6 (Supplemental Figures S5, S6). Furthermore, M4 exhibits substantial changes in its pattern when bS21 is present, which is consistent with the fact that this construct exhibits low but detectable protein binding levels in the dot-blot assays (Figure 3C). Overall, these results indicate that the two base-pair transversions that define M7 (Figures 3B, 4A) permit the formation of a WT-like RNA structure, but the resulting RNA fails to be strongly bound by bS21. This is consistent with our hypothesis that the protein binding determinants are certain nucleotides within the SD-ASD mimic region. Thus, the loss of bS21 affinity for the mutant constructs is not due exclusively to secondary structure misfolding, but rather is dependent on a specific nucleotide sequence or perhaps a local, fine structure of OLE RNA.

### Binding of bS21 induces formation of a k-turn substructure in OLE RNA

The original secondary structure model proposed for OLE RNAs [3] was based on comparative sequence analysis and nucleotide covariation, which provides strong support for the existence of base-paired stems (including P5 and P6) as also depicted in the updated model (Figure 1). Covariation analysis is a powerful predictor of secondary structure features because nucleotide changes through evolution retain important Watson-Crick base-pairing potential [35]. Indeed, in-line probing results supporting the formation of P5 and P6 by protein-free OLE RNA have been reported previously [12,16], and these results are confirmed herein (Figure 4). However, upon OapC binding, a conformational change occurs involving formation of a k-turn structure (here called k-turn 1a) that disrupts some of the phylogenetically conserved base-pairs of P6 [16]. Subsequent to the biochemical detection of the k-turn 1a substructure, formed by interactions between J4a/5, J5/6, and parts of P6, we also identified [16] nucleotide covariation evidence for this same structure (Figure 1, left inset).

In a recent report describing a partial OLE RNP structure model based on cryo-EM data [36], a third, mutually exclusive structure (here called k-turn 1b) is proposed to form by J4a/5 and J6/7. Intriguingly, there is also covariation evidence supporting this substructure (Figure 1, right inset). Thus, biochemical, biophysical, and comparative sequence analyses indicate that the J4a/5 to J6/7 region encompassing the P5 and P6 stems can form at least three distinct but biologically relevant structures, i.e. P5 and P6 with J5/6, k-turn 1a, and k-turn 1b (Supplemental Figure S1). This small region presumably also must participate in the formation of the larger OLE RNA tertiary structure.

**Figure 5.**
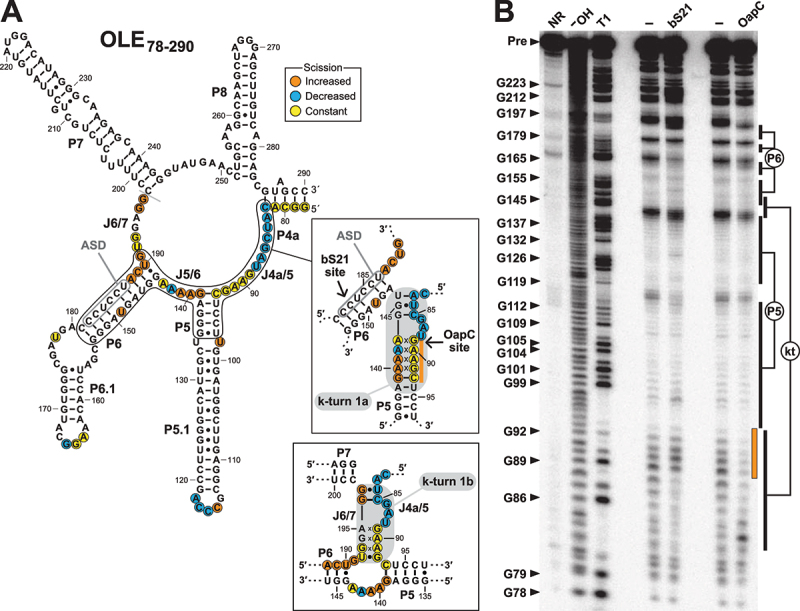
In-line probing assays of 5' ^32^P-labelled OLE_78–290_ RNA. (A) Sequence and secondary structure model for OLE_83–191_ RNA. Insets depict alternative structures that include the formation of k-turn 1a and k-turn 1b. In-line probing characteristics are mapped for the data derived with bS21 as depicted in B. Note that the scission information derived from B is more consistent with formation of k-turn 1a and not k-turn 1b. Other annotations are as described for Figure 4A. (B) Autoradiogram of in-line probing assays for 5' ^32^P-labelled OLE_78–290_ RNA in the absence (‒) or presence of 1 µM bS21 or 2 µM OapC as indicated. The orange bar (see also the inset in A) identifies differences in band intensities between in-line probing assays conducted with bS21 and OapC, consistent with OapC binding the k-turn structure. Other annotations are as described for Figure 4B.

The evidence for three mutually exclusive structures in this small region of OLE RNA highlights the possibility that bS21 binding might trigger structural changes involving these additional structural states. To address this possibility, in-line probing was used to evaluate the structure of the WT 5' ^32^P-labelled OLE_78–290_ construct (Figure 5A) in the absence and presence of bS21. This larger construct was chosen for this analysis so that the J6/7 region that participates in formation of k-turn 1b [36] is included. In the absence of bS21, the OLE_78–290_ RNA fragment exhibits a pattern of spontaneous RNA cleavage products (Figure 5B) that are again consistent with the formation of the P5 and P6 stems as originally predicted for OLE RNAs [3,13]. Inclusion of 1 µM bS21 results in a pattern of spontaneous RNA cleavage products that is consistent with k-turn 1a formation, and is similar to the pattern observed when 2 µM OapC is present. Thus, it appears that bS21 and OapC induce the formation of highly similar alternative structures. The fact that bS21 induces a conformational change similar to that for a validated partner of the OLE RNP complex supports the hypothesis that bS21 is also a biologically relevant component of this particle.

Despite the similarities between the in-line probing datasets for bS21 and OapC, there are notable differences. For example, the extent of strand scission at nucleotides 89 through 92 in J4a/5 is unchanged when bS21 is added, but is substantially decreased with OapC. This can be explained if OapC binds and stabilizes the structures involving these nucleotides within the 5' portion of the k-turn, as is expected from a published structure of a similar k-turn binding protein interacting with its RNA target [22]. In contrast, if bS21 binds OLE RNA at nucleotides localized in the P6 region as we predict, this protein is not likely to directly protect nucleotides 89 through 92 from spontaneous cleavage. The addition of OapC also results in the suppression of strand scission at nucleotides 193–195, whereas bS21 does not affect this region of the RNA. Because it has been proposed that OapC stabilizes the folding of either k-turn 1a [16] or k-turn 1b [36], and both share the same 5' portion (Supplemental Figure S1), the in-line probing data with OapC might reflect two distinct folds of OLE_78–290_ RNA. The addition of bS21 appears to favour formation of k-turn 1a, but not k-turn 1b (Figure 5B).

The proposed mechanism of action of bS21 in translation initiation is to melt a long-distance intramolecular interaction within the 16S rRNA to free the 3' terminus of the 16S rRNA containing the ASD sequence [28]. This 3' terminus is single stranded in active ribosomes [37,38] but not in inactive ribosomes [39]. Notably, bS21 binding increases spontaneous cleavage of RNA linkages near the 3' end of P6 (involving nucleotides 188–191). These same nucleotides become unpaired upon formation of k-turn 1a (Supplemental Figure S1). These results support our hypothesis that bS21 promotes melting of a portion of P6 and subsequent formation of k-turn 1a. However, it is also possible that bS21 binding might permit OLE_78–290_ to toggle between the two k-turn substructures, thereby providing OapC with a choice of k-turn substructures that could be bound. Thus, bS21 and OapC might be co-resident on the same OLE RNA. The two recent cryo-EM partial structural models of OLE RNA [36,40] reveal that there is sufficient space for the simultaneous binding of bS21 and OapC, even though their bindings sites are near each other in 3D space. Furthermore, both models propose OLE RNAs form a homodimer with intermolecular interactions between the P4 loops of each monomer, between the loop of P5 of one monomer and the loop of P6 of the other (twice), and another between the P9 loops of each monomer. The decrease in spontaneous cleavage of nucleotides of L5 and L6 upon binding of bS21 (Figure 4) raises the exciting possibility that bS21 might indirectly facilitate this loop–loop interaction and ultimately favour dimerization of OLE RNA, which merits future investigation.

### Matching growth deficiency phenotypes caused by disruptive mutations to bS21 or its OLE RNA binding site

The results described above, along with the results of a previously reported RNA pull-down study [16], provide strong support that bS21 is a biologically relevant component of the OLE RNP complex. To further assess this hypothesis, we examined the growth characteristics of WT and genetically altered *H. halodurans* strains that lack OLE RNA, a functional bS21, or that carry disruptive mutations at the bS21 binding site of OLE RNA. These strains were subjected to spot assays on agar media under optimal conditions or under ethanol (5.5% v/v), cold (18°C), Mg^2+^ (10 mM), or starvation (no glucose) stress conditions.

**Figure 6.**
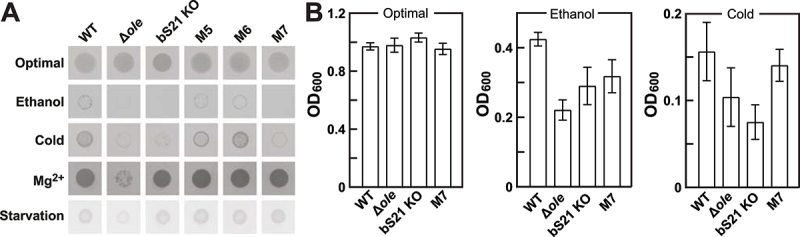
Stress phenotypes of a bS21 knockout strain overlap with a strain carrying a mutant OLE RNA that fails to bind bS21. (A) Agar spot assays with *H. halodurans* wild-type (WT), Δ*ole*, bS21 KO cells or cells carrying OLE RNA mutants M5, M6, or M7. Cells were subjected to either no stress (optimal) or to ethanol (5.5% v/v), cold (18°C), Mg^2+^ (10 mM), or a non-glucose carbon source (starvation, glutamate carbon/energy source) stresses. Bacterial cultures were diluted to an OD_600_ of 0.5 (or 0.005 for ethanol stress) and spotted onto agar media with the appropriate compositions (see materials and methods). Plates were incubated for 24 h (optimal, Mg^2+^), 48 h (ethanol, starvation) at 37°C, or for 6 days at 18°C for cold stress. The images depict representative assays from three biological replicates each with three technical replicates. (B) Liquid culture growth assays with *H. halodurans* WT, Δ*ole*, bS21 KO, or OLE RNA M7 strains under optimal conditions (LB medium, pH 10), ethanol stress (5% [v/v]), or cold stress (18°C). Initial cultures were diluted to an OD_600_ of 0.01 and grown at 37°C (optimal and ethanol stress conditions) with 220 rpm shaking for 24 h (optimal) or 25 h (ethanol stress). Cultures for cold stress were incubated at 18°C with 220 rpm shaking for 72 h. Bars represent the average OD_600_ values from three biological replicates with three technical replicates each. Error bars represent standard deviation from the mean.

All strains exhibited robust growth under optimal medium conditions (LB medium, pH 10, 37°C), in the absence of any of the stress conditions examined (Figure 6A). Substantial growth of WT cells was observed under all stress conditions, whereas cells lacking the *ole* gene (∆*ole*) exhibited little or no detectable growth when subjected to any of the four stresses, matching the phenotypes reported previously [9–11] for cells lacking a functional OLE RNP complex. Although the strain with a functional bS21 KO (Glu3*, Arg5*; asterisk indicates a stop codon) grows robustly under optimal conditions, it matches the growth of WT cells only under Mg^2+^ and starvation stresses. This strain fails to grow well under ethanol or cold stresses. The observation that the bS21 KO strain does not perfectly phenocopy the ∆*ole* strain indicates that it is not an essential component of the OLE RNP complex for its general function, like OapA [9], OapB [13], and OapC [16].

If the two stress phenotypes exhibited by the bS21 KO strain are due to its absence in the OLE RNP complex, then OLE RNA mutants that fail to bind the protein might also share the same phenotype pattern. To address this hypothesis, we generated OLE RNA mutant strains M5, M6 and M7 in *H. halodurans* by introducing mutations into the endogenous *ole* gene. These mutations were chosen for testing because this series exhibited progressively poorer bS21 binding characteristics (Figure 3D). Strains carrying either the M5 or M6 versions of OLE RNA exhibit WT-like growth under all stress conditions, suggesting either that the reduction of bS21 binding affinity for these two OLE RNA mutants is either insufficient to cause a phenotype or that this interaction is not relevant to the observed phenotypes. However, these results do indicate that mutations to the highly conserved G-C base-pairs in the SD-ASD mimic region do not destroy the general function of the OLE RNP complex.

Intriguingly, the bacterial strain carrying the M7 OLE RNA construct exhibits the same stress phenotype distribution as cells lacking bS21 (Figure 6A). This same correlation is largely reproduced when these strains are grown in liquid media under optimal conditions, or under ethanol or cold stresses (Figure 6B). The M7 version of OLE_83–191_ consistently ranked as the poorest RNA target for bS21 binding (Figure 3D), which might explain why cells carrying the M7 version of OLE RNA match the phenotype pattern of bS21 KO cells, whereas M5 and M6 exhibit WT-like growth patterns.

## Conclusions

Since the initial discovery of OLE RNA two decades ago [3], several findings highlight the intricate structural and functional features of this large ncRNA and the particle it forms. OLE RNA appears to be the central component of an RNP complex that plays fundamental roles in bacterial stress sensing and adaptation [1]. On the growing list of physical or functional connections to OLE RNA is the process of protein translation. Previously [1], it was noted that two genes (*nusB* and *yqxC*) relevant to ribosome biosynthesis are commonly located near *ole* and *oapA* genes, suggesting their functions are somehow related. In addition, it was shown [11] that disruption of the protein BmrC, which forms a putative multidrug transporter together with BmrD [41], suppresses the growth deficiency phenotype observed under cold, Mg^2+^, and non-preferred carbon/energy source stresses for cells whose OLE RNP complex has been functionally disrupted. In *B. subtilis*, these two proteins are known to be expressed only when translation of an upstream open reading frame (uORF) in the *bmrCD* operon is slowed [42]. Thus, the initial observation that ribosomal protein bS21 is a possible partner of the OLE RNP complex [16] fits with these previous findings, and together increase the likelihood that the two macromolecules form a biologically relevant complex.

The findings described herein demonstrate that OLE RNA binding by bS21 is biologically relevant. *H. halodurans* bS21 selectively binds OLE RNA in a region of P6 that mimics the SD-ASD interaction between mRNAs and 16S rRNAs. The presence of this OLE RNA substructure and its interaction with bS21 could simply have occurred by chance, and their cross-linking in OLE RNA pull-down experiments [16] could also have been incidental. However, several observations indicate that the two molecules form a natural, biologically important partnership. For example, bS21 binding to OLE RNA induces a structural change that is very similar to that required for the binding of OapC, and essential protein partner for OLE RNP complex function. Such protein-induced structural changes can highlight the complexity of OLE RNA because they provide evidence of sophisticated structure switching as proteins engage or depart the RNP complex. In addition, identical patterns of stress phenotypes are observed when either bS21 is disabled or when OLE RNA carries mutations that most strongly disrupt bS21 binding. Notably, mutation of highly conserved nucleotides in the SD-ASD structure can diminish or eliminate bS21 binding without destroying the broader functions of the OLE RNP complex. Likewise, the absence of functional bS21 does not entirely disable the complex, and therefore this protein is not an essential component for all its functions.

From these findings, we conclude that bS21 is a natural component of a form of OLE RNA complex that is involved in physiological adaptation to stresses caused by exposure to ethanol (and likely other short-chain alcohols) and to low temperatures. Thus, bS21 has an extraribosomal or ‘moonlighting’ function that might support OLE RNA folding and function, and/or play an active role in integrating the status of translation with OLE RNP stress response activities. We have proposed [1] that OLE RNP complexes perform many diverse functions necessary for cells to manage various stresses. This might best be achieved by forming dynamic RNP particles that change shape, dock and undock various proteins in response to different stresses, and interface with fundamental cellular processes to enable major biochemical and physiological responses to occur. The broad, cell-wide control of gene expression is an obvious means to make major changes in response to fundamental stresses.

The results from the current study provide a physical link between OLE RNA and the process of translation. During ribosome assembly, bS21 is the last ribosomal protein to join the complex [43–45]. Bacterial species that mostly encode leaderless mRNAs (mRNAs lacking an SD sequence) tend to lack bS21 [46], suggesting that this protein is required for efficient translation of mRNAs with leader sequences. In the context of the assembled ribosome, bS21 has been shown to be most important for mRNA binding among the ribosomal proteins [47]. Because bS21 affects SD-ASD interactions between 16S rRNAs and mRNAs, the protein can be expected to affect the efficiency of translation initiation as a mechanism of cell-wide translation control [26–29]. Indeed, evidence continues to emerge that bS21 is an active component in the selection of mRNAs for translation [48–50]. Furthermore, bS21 is loosely bound to the ribosome and exchange of bS21 among ribosomes in vivo has been observed [51,52]. These characteristics could be exploited by the OLE RNP complex to sense disturbances in translation via monitoring the levels of free bS21 (not associated with ribosomes), or perhaps to actively adjust the pool of bS21 proteins available for use by ribosomes in response to specific stresses sensed by the OLE RNP complex. In other words, broad changes in ribosome activity could affect the composition and function of OLE RNP, or vice versa.

It is important to note that ribosomal proteins bS1, uS7, and uS11 also appear as candidates for interaction with OLE RNA based on the RNA ‘pull-down’ study that revealed bS21 as a candidate [16]. In *B. subtilis* ribosomes, uS7 and uS11 are known [53] to bind adjacent to bS21, and therefore might similarly cluster in OLE RNP complexes. Whether bS1 is part of the ribosome in Gram-positive bacteria is less clear due to the absence of domains involved in rRNA binding and binding of other ribosomal proteins [38] normally present in the *E*. *coli* protein [25]. In addition, we have preliminary evidence that YqeY is a protein binding partner of OLE RNA [1] (Supplemental Figure S7). The genes for YqeY and bS21 often reside in an operon, suggesting that they have functional roles that are related. If these potential connections prove true, then OLE RNA will have additional physical interactions with moonlighting proteins that are otherwise only known to be relevant to the process of translation.

Despite numerous findings regarding the biological roles of OLE RNA [1], the precise molecular mechanisms of action of this RNA remain elusive. Identification of additional protein partners, especially those with known biological and biochemical functions, would greatly benefit the effort to identify these mechanisms. Numerous additional protein partner candidates for OLE RNA, derived from bioinformatic [20], genetic [10,11,13], and biochemical [16] approaches, remain to be experimentally evaluated. Some of these candidates might help reveal new biological processes engaged by the OLE RNP complex, and help explain how the complex assembles at, or even protrudes through [36], the lipid bilayers of their host cells.

## Materials and methods

### Bioinformatic analyses

The updated consensus model for the OLE RNA class was generated as previously described [13] using the prior alignment of 798 OLE RNAs [1] as a seed file in a search against RefSeq [54] version 217 and environmental DNA sequences as previously described [55]. Covariation analyses of both k-turn variants in OLE RNA were completed by manual assessment of the OLE RNA alignment. R2R [56] was used to calculate and represent covariation significance. The respective sequence data files in Stockholm format are provided (Supplemental Data Files ole_refseq217_env12.sto, ole_kturn_1a_refseq217_env12.sto and ole_kturn_1b_refseq217_env12.sto).

### RNA preparation

DNA templates for in vitro transcription that contain the promoter sequence for T7 RNA polymerase (RNAP) were generated by PCR. Synthetic oligonucleotide primers and PCR templates are listed in Table 1. Subsequent in vitro transcription reactions, RNA purifications, and 5' ^32^P-labelling reactions were performed as previously described for OLE RNA and its segments [12].

**Table 1.** Oligonucleotides. Bold Gs indicate G nucleotides added to natural OLE sequence for more efficient in vitro transcription, underlined sequences indicate T7 promoter sequence, lowercase letters indicate point mutations introduced.

Purpose		Template		Name		Sequence
Verification of successful cloning and transformation						
		pBASE-derived vectors		pBASE6_MCS_F		GATGCCTCAAGCTAGAGAGTCATTACC
				pBASE6_MCS_R		CCATGTATTCACTACTTCTTTCAAACTCTCTC
		pHCMC05-derived vectors		pHCMC05-FWD		GTTGTTGACTTTATCTACAAGGTGTGGC
				pHCMC05-REV		CCAGGTAAGGTATAAACTTTTCAGTTGC
Cloning pHCMC05-ole-oapA_P6_M1 to M7 (pFW018–021, pFW023, 025, 024)						
M1 to M7		pHCMC05-ole-oapA wt		FW039 + (1)		GGCGAGTTACATGATCCCCCATGTTGT
				FW040 + (2)		GGGGGATCATGTAACTCGCCTTGAT
M1 (pFW018)		pHCMC05-ole-oapA wt		(1) FW137		CACAAAGGCATGTGGGCGATGACCCaCCTACT
				(2) FW138		CCCACATGCCTTTGTGGATCGCCCaACTACCT
M2 (pFW019)		pHCMC05-ole-oapA wt		(1) FW139		CAAAGGCATGTGGGCGATGACCCaCCaACTGTGGA
				(2) FW140		CCCACATGCCTTTGTGGATCGCCCaACaACCTTT
M3 (pFW020)		pHCMC05-ole-oapA wt		(1) FW141		CAAAGGCATGTGGGCGATGACCCatCaACTGTGGA
				(2) FW142		CCCACATGCCTTTGTGGATCGCCCagCaACCTTT
M4 (pFW021)		pHCMC05-ole-oapA wt		(1) FW143		CAAAGGCATGTGGGCGATGACgCatCaACTGTGGA
				(2) FW144		CCCACATGCCTTTGTGGATCGCgCagCaACCTTT
M5 (pFW023)		pHCMC05-ole-oapA wt		(1) FW145		CAAAGGCATGTGGGCGATGACgCTCCTACTGTGGA
				(2) FW146		CCCACATGCCTTTGTGGATCGCgCTACTACCTTT
M6 (pFW025)		pHCMC05-ole-oapA wt		(1) FW149		CAAAGGCATGTGGGCGATGACCgTCCTACTGTGGA
				(2) FW150		CCCACATGCCTTTGTGGATCGCCgTACTACCTTT
M7 (pFW024)		pHCMC05-ole-oapA wt		(1) FW147		CAAAGGCATGTGGGCGATGACggTCCTACTGTGGA
				(2) FW148		CCCACATGCCTTTGTGGATCGCggTACTACCTTT
Cloning pBASE_Bha_ole +1kb_fl (pFW022)						
		gDNA *H. halodurans*		FW151		GTGCAGCGGAAGAACTGTGTCCCGTTTCCCAAA
				FW152		GGCATGCAAATCAGATATTGAAAGAAGATGGGAA
		pFW004		FW153		GGACACAGTTCTTCCGCTGCACTGCGATGAGT
				FW039		GGCGAGTTACATGATCCCCCATGTTGT
				FW154		CAATATCTGATTTGCATGCCTGCAGAACGGATTGTTG
				FW040		GGGGGATCATGTAACTCGCCTTGAT
Cloning pBASE_Bha_ole +1kb_fl_P6_M5 (pFW028)						
		pFW022		FW145		CAAAGGCATGTGGGCGATGACgCTCCTACTGTGGA
				FW039		GGCGAGTTACATGATCCCCCATGTTGT
				FW146		CCCACATGCCTTTGTGGATCGCgCTACTACCTTT
				FW040		GGGGGATCATGTAACTCGCCTTGAT
Cloning pBASE_Bha_ole +1kb_fl_P6_M6 (pFW030)						
	pFW022		FW149		CAAAGGCATGTGGGCGATGACCgTCCTACTGTGGA	
			FW039		GGCGAGTTACATGATCCCCCATGTTGT	
			FW150		CCCACATGCCTTTGTGGATCGCCgTACTACCTTT	
			FW040		GGGGGATCATGTAACTCGCCTTGAT	
Cloning pBASE_Bha_ole +1kb_fl_P6_M7 (pFW029)						
	pFW022		FW147		CAAAGGCATGTGGGCGATGACggTCCTACTGTGGA	
			FW039		GGCGAGTTACATGATCCCCCATGTTGT	
			FW148		CCCACATGCCTTTGTGGATCGCggTACTACCTTT	
			FW040		GGGGGATCATGTAACTCGCCTTGAT	
PCRs to generate T7 templates for in vitro transcription						
OLE_1–637_	pHCMC05-ole-oapA wt		KH013		TAATACGACTCACTATA**GG**TGTCTTTTAGAATAAGAGTGG	
			KH014		CGTTCCGACTGCGTATGTATGA	
OLE_78–290_	pHCMC05-ole-oapA wt		KH189		TAATACGACTCACTATA**GG**GGCACATCGATGAAGCTCCTTG	
			KH190		GGCTACGCTGCTGACAAGC	
OLE_83–191_	pHCMC05-ole-oapA wt		FW030		TAATACGACTCACTATA**GG**ATCGATGAAGCTCCTTGTGATGGCTTGA	
			FW033		CAGTAGGAGGGTCATCGCCCACAT	
OLE_139–284_	pHCMC05-ole-oapA wt		FW129		TAATACGACTCACTATA**GG**AAAAGGTAGTAGGGCGAT	
			FW031		GCTGCTGACAAGCTCCCTAGCTTGCCTT	
OLE_191–284_	pHCMC05-ole-oapA wt		FW130		TAATACGACTCACTATA**GG**TGGAGGCCTTTTTCTCTGCT	
			FW031		GCTGCTGACAAGCTCCCTAGCTTGCCTT	
OLE_449–608_	pHCMC05-ole-oapA wt		KH193		TAATACGACTCACTATA**GG**CATTTAAAGAGGATTACAGTGTGGACTAAGTG	
			KH194		CATTTAAAGAGTAAACTCTGTGGCTTAGGTCC	
OLE_83–191_ M1, M2, M3, M4, M5, M6, M7	pFW018–021, 023, 025, 024		FW030		TAATACGACTCACTATA**GG**ATCGATGAAGCTCCTTGTGATGGCTTGA	
			FW033		CAGTAGGAGGGTCATCGCCCACAT	

### Overexpression and purification of bS21

The construct for expression of the bS21 protein was designed with an N-terminal hexa-histidine tag followed by a PreScission protease cleavage site. The Codon Optimization Tool from IDT DNA (Coralville, IA, USA) was used to adjust the *H. halodurans* gene sequence for optimal expression in *E. coli*. The DNA insert of the His6-bS21 (*rpsU*, AYT26_RS07045) template was synthesized and cloned into pET-11a at NdeI and BamHI sites by GenScript (Piscataway Township, NJ, USA). The plasmid was transformed into *E*. *coli* BL21 Star (DE3) (C601003, Thermo Fisher Scientific, Fair Lawn, NJ, USA) according to the manufacturer’s instructions. Transformants were confirmed by colony PCR followed by DNA sequencing of the amplified product.

For bS21 protein expression, a single colony of the transformed strain was used to inoculate 10 mL of Luria Bertani (LB) medium supplemented with 100 μg mL^−1^ carbenicillin. The mixture was incubated overnight with shaking at 37°C and the resulting culture was used to inoculate 1 L of identical medium. The culture was incubated with shaking at 37°C until the absorbance at 600 nm (OD_600_) attained a value between 0.4 and 0.6. Protein expression was then induced by addition of isopropyl β-D-1-thiogalactopyranoside (IPTG) to a final concentration of 0.5 mM. The induced mixture was incubated overnight at 16°C with shaking. Cells were harvested by centrifugation at 4°C for 20 min at 6,000 g. Pelleted cells were resuspended in 50 mL phosphate-buffered saline (PBS), split into two aliquots and harvested by centrifugation at 4°C for 5 min at 10,000 g. Pelleted cells were frozen at −80°C until subjected to bS21 purification.

Protein purification was conducted with one aliquot of pelleted cells by thawing on ice for 1 h, then resuspending the cells with 5 mL of cell lysis buffer (50 mM Tris-HCl [pH 7.5 at 20°C], 1 M NaCl, 20 mM imidazole, EDTA-free protease inhibitors [1 tablet per 50 mL, A32965, Thermo Fisher Scientific]) per gram of cell pellet. Cells in this mixture, kept on ice, were lysed by sonication. Non-lysed cells and debris were removed by centrifugation at 4°C for 30 min at 16,000 g. The resulting supernatant was transferred to a new tube that was stored on ice. Cell-free lysate was then applied to a pre-equilibrated (50 mM Tris-HCl [pH 7.5 at 20°C], 500 mM NaCl) Ni-NTA agarose column (R90115, Invitrogen, Thermo Fisher Scientific) (2 mL bed volume) using gravity flow. The column was washed with 20 column volumes of wash buffer (50 mM Tris – HCl [pH 7.5 at 20°C], 1 M NaCl, 20 mM imidazole), and then eluted with 5 column volumes elution buffer (50 mM Tris-HCl [pH 7.5 at 20°C], 1 M NaCl, 500 mM imidazole).

The eluent was concentrated with an MilliporeSigma™ Amicon Ultra-4 Centrifugal Filter Unit (UFC80030, Thermo Fisher Scientific) with a molecular weight cut-off of 3 kDa. Imidazole was removed using a PD-10 desalting column with Sephadex G-25 resin (#17085101, Cytiva, Marlborough, MA, USA) equilibrated in buffer (50 mM Tris-HCl [pH 7.5 at 20°C], 1 M NaCl) following the manufacturer’s instructions for gravity elution. N-terminal hexa-histidine tag was removed by incubation with Pierce HRV 3C protease (1 mg mL^−1^, 2 U μL^−1^, #88946, ThermoScientific) overnight at 4°C under gentle agitation. The concentration of NaCl in the protease reaction was reduced to 500 mM and bS21 was further purified using a pre-equilibrated Ni-NTA agarose column and concentrated as described above. Protein purity and efficient hexa-histidine tag removal was assessed by SDS-PAGE with Coomassie staining and visualized using ChemiDoc MP imaging system (Bio-Rad, Hercules, CA, USA) (Supplemental Figure S2). Protein concentrations were determined using a standard Bradford Assay. Purified samples were aliquoted, flash-frozen with liquid nitrogen, and stored at −80°C. OapC was purified as previously described [16].

### Filter-binding assays with bS21

Filter-binding assays were performed using a 96-well dot-blot filter apparatus (Bio-Rad) with both nitrocellulose (0.45 μm, #10600012, Cytiva) and nylon (0.45 μm, # RPN303B, Cytiva) membranes positioned in the filtration liquid flow in series. Prior to assembly, the membranes were pre-equilibrated by soaking in deionized water (dH_2_O) (two exchanges) followed by binding buffer (20 mM Tris-HCl [pH 7.5 at ~20°C], 150 mM NaCl, and 10 mM MgCl_2_). After assembling the apparatus with pre-equilibrated membranes, each well was washed with 30 μL binding buffer. Binding assays (15 μL final volume) were performed using ~2.5 nM 5' ^32^P-labelled RNA, exhibiting ~500 counts per minute (cpm), and bS21 in binding buffer supplemented with 1 mM tris(2-carboxyethyl)phosphine (TCEP), 5% (v/v) glycerol, and 0.001% (w/v) SDS (final concentrations). Glycerol was included to enable aliquots of the reactions to be loaded and analysed by polyacrylamide gel electrophoresis (PAGE). SDS was added to reduce non-specific adherence of RNA to surfaces [57].

Assays were assembled at 20°C and incubated for 15 min before loading onto the dot-blot apparatus. After filtration, each well was washed once with 30 μL binding buffer. The apparatus was then disassembled, and the membranes were dried with a blow dryer before being imaged using a phosphorimager (GE Healthcare, Chicago, IL, USA). The resulting band intensities were quantified using ImageQuant TL 8.1 (GE Healthcare). The binding data for each experiment were normalized by first subtracting the amount of radioactivity measured on the nitrocellulose membrane in the absence of protein from the values recorded for all other incubations. The background value was also added to the radioactivity measured for each nylon membrane blot. These corrections assume that the RNA adhered to the nitrocellulose membrane actually represents free RNA, which is a known artefact [58]. Percentage bound values were then computed by dividing the counts of bound RNA by the total counts (bound plus free RNA) and multiplying by 100. The average normalized percentage bound values and standard deviation (SD) values were calculated from technical replicates (*n* = 3). Note that variability in percentage bound values can occur between experiments, perhaps in part due to experimental variables such as the vacuum strength or reagent preparations used. As a result, we did not attempt to establish precise *K*_D_ values using this method.

### In-line probing assays

5' ^32^P-radiolabelled OLE RNA constructs defined for each in-line probing assay [33,34] experiment at ~150 nM (~10,000 cpm) were refolded by incubation in dH_2_O at 75°C for 1 min followed by cooling to ~20°C over 5 min. Unlabelled OLE_449–608_ RNA (not bound by bS21 or OapC) was added to a final concentration of 1 μM to serve as a decoy for RNase contaminants that might be present in the protein samples. This precaution was expected to decrease RNase degradation of the 5' ^32^P-radiolabelled OLE RNA that would otherwise obscure the banding pattern generated by spontaneous RNA cleavage. Reactions also contained a final concentration of 50 mM Tris-HCl (pH 8.3 at ~20°C), 20 mM MgCl_2_, and 100 mM KCl. Samples were incubated in the absence or presence of bS21 or OapC as indicated for each experiment at ~20°C for 40 h. The reactions were quenched with an equal volume of loading solution (8 M urea, 20% [w/v] sucrose, 0.1% [w/v] SDS, 0.05% [w/v] bromophenol blue, 0.05% [w/v] xylene cyanol, 90 mM Tris base, 90 mM borate, and 1 mM EDTA) and then subjected to denaturing (8 M urea) 10% PAGE. Gels were dried, imaged by phosphorimager as described above.

### Bacterial assays

All strains and plasmids used in this study are listed in Table 2. Chemically competent *E. coli* cells were transformed using a standard heat shock procedure following the general protocol described previously [59]. *H. halodurans* was transformed via protoplast transformation [7] and chromosomal modifications (insertion of point mutations) were made as described previously [8]. All *H. halodurans* C-125 (JCM 9153) strains were grown in Luria-Bertani (LB) medium (Ready-Made LB Broth Powder [J75852-A1, Thermo Fisher Scientific]) containing 1% (w/v) sodium carbonate hexahydrate such that the medium was pH 10. Cultures were grown at 37°C with shaking at 220 rpm if not stated otherwise.

**Table 2.** Bacterial strains and plasmids.

Strains	Description	Reference
*E. coli* BL21 Star (DE3)	Commercially available T7 expression *E. coli* strain, deficient in proteases Lon and OmpT, resistant to phage T1 (fhuA2), increased mRNA stabilities due to RNase E mutation, common strain for protein expression	Thermo Fisher Scientific C601003
*E. coli* TOP10 + pPAMC125	*E. coli* TOP10 (commercially available) with pPAMC125 vector for in vivo methylation of plasmids to be transformed into *H. halodurans* C-125	[7]
*H. halodurans* C-125 (‘Bha’)	Rod-shaped, Gram-positive, motile and spore-forming, alkaliphile, halophile, and originally isolated from soil	[5,6]
*H. halodurans* C-125 Δ*ole*	Deletion of *ole*	[9]
*H. halodurans* C-125 rpsU**	Functional bS21 knockout strain (rpsU**: insertion of two early stop codons, Glu3* and Arg5* in *rpsU*)	[8]
*H. halodurans* C-125 *ole* M5	G152C and C182G point mutations in *ole*	This study
*H. halodurans* C-125 *ole* M6	G151C and C183G point mutations in *ole*	This study
*H. halodurans* C-125 *ole* M7	G151C, G152C, C182G and C183G point mutations in *ole*	This study
Plasmids		
pET-11a_FW_rpsU_Bha (pFW002)	ori pBR322, *bla*, *lacI*, T7 promoter with *lac* operator, His6-bS21	This study
pBASE_Bha (pFW004)	ori pE194ts (temperature-sensitive); ori ColE1; G+/G- shuttle; *cat*, *bla*; Bha antisense *secY* under P_xyl/tet_ promoter	[8]
pBASE_Bha_ole +1kb_fl (pFW022)	ori pE194ts (temperature-sensitive); ori ColE1; G+/G- shuttle; *cat*, *bla*; Bha antisense *secY* under P_xyl/tet_ promoter, *ole* gene with 1 kb flanking regions	This study
pFW028	pFW022 with ‘*ole* M5’ G152C, C182G	This study
pFW029	pFW022 with ‘*ole* M7’ G151C, G152C, C182G, C183G	This study
pFW030	pFW022 with ‘*ole* M6’ G151C, C183G	This study
pHCMC05-ole-oapA wt	Theta circle replication, G+/G- shuttle; *cat*, *bla*; *ole-oapA* under IPTG-inducible promoter	[9]
pHCMC05-ole-oapA_P6_M5 (pFW023)	‘*ole* M5’ G152C, C182G	This study
pHCMC05-ole-oapA_P6_M6 (pFW025)	‘*ole* M6’ G151C, C183G	This study
pHCMC05-ole-oapA_P6_M7 (pFW024)	‘*ole* M7’ G151C, G152C, C182G, C183G	This study

For liquid cultures, bacteria were first grown overnight in 3 mL of LB (pH 10). The next morning, OD_600_ values of the overnight cultures were measured using a Cary 60 UV-Vis Spectrophotometer (Agilent Technologies, Santa Clara, CA, USA), and the cultures were then diluted to an OD_600_ of ~ 0.05 in 3 mL of fresh LB (pH 10) medium and grown for 3 to 4 h. Next, the OD_600_ of the cell cultures was measured (usually 0.5–0.8) and the cells were then diluted to an OD_600_ of 0.01 in the designated media: LB (pH 10) (optimal), LB (pH 10) with 5.5% [v/v] ethanol (ethanol stress), or LB (pH 10) with incubation at 18°C (cold stress). Growth assays were performed with 3 mL of media in 14 mL culture tubes. For optimal and ethanol stress conditions, cultures were incubated at 37°C with shaking at 220 rpm for 24 and 25 h, respectively. Cultures for cold stress were incubated at 18°C with shaking at 220 rpm for 72 h. At the designated time point, 200 µL of these cultures was aliquoted into wells of a clear, flat-bottom 96-well plate, and the OD_600_ was measured using a BioTek Synergy Neo2 Multimode reader. Unless stated otherwise, all growth assays were performed in biological triplicates wherein each biological replicate was comprised of three technical replicates.

Agar spot assays were conducted by first culturing bacteria overnight in 3 mL of LB (pH 10). OD_600_ values of the overnight cultures were measured as described above, and the cultures were then diluted with PBS to an OD_600_ of 0.5 for most assays, or 0.005 for ethanol stress assays. A total of 2.5 μL of diluted overnight cultures were then spotted onto agar in square plates (15 × 100 × 100 mm, MilliporeSigma™ Petri Dishes Specialty, #FB0875711A, Thermo Fisher Scientific). When the media component of the agar was either LB (pH 10) (for optimal and cold stress) or LB (pH 10) supplemented with 5.5% [v/v] ethanol (ethanol stress) or 10 mM MgCl_2_ (Mg^2+^ stress), the agar concentration was 1.5% [w/v]. For glutamate minimal media (EMM) agar (starvation stress), Noble Agar (Ultrapure, J10907.36, Thermo Fisher Scientific) at a final concentration of 0.6% [w/v] was used. EMM was modified from TSS medium [60] to permit growth of *H. halodurans*. EMM contained 100 mM sodium bicarbonate (pH 8.5 at ~20°C), 2.5 mM K_2_HPO_4_, 0.2% [w/v] NH_4_Cl, 0.02% [w/v] MgSO_4_-heptahydrate, 40 µg mL^−1^ FeCl_3_-hexahydrate, 40 µg mL^−1^ sodium citrate-dihydrate, and 20 mM glutamate. Agar plates were incubated for 24 h (optimal and Mg^2+^ stress), 48 h (ethanol stress and starvation stress) at 37°C or for 6 days at 18°C. For ethanol stress, outermost squares of agar were not used for spotting due to inconsistent results likely caused by increased ethanol evaporation in these regions. Images were acquired using the GelDoc Go Imaging System (Bio-Rad) with ‘stain-free gel’ settings.

## Supplementary Material

Supplemental Material

## Data Availability

All data for this study is presented in the main text, supplemental figures, or supplemental data files. Any further underlying data will be made available upon reasonable request from the corresponding author.
